# Pattern of Investigation Reflects Risk Profile in Emergency Medical Admissions

**DOI:** 10.3390/jcm4051113

**Published:** 2015-05-21

**Authors:** Seán Cournane, Declan Byrne, Deirdre O’Riordan, Niall Sheehy, Bernard Silke

**Affiliations:** 1 Department of Medical Physics and Bioengineering, St James’s Hospital, Dublin 8, Ireland; E-Mail: scournane@stjames.ie; 2 Department of Internal Medicine, St James’s Hospital, Dublin 8, Ireland; E-Mails: declangbyrne@gmail.com (D.B.); doriordan@stjames.ie (D.O.R.); 3 Diagnostic Imaging Department, St. James’s Hospital, Dublin 8, Ireland; E-Mail: nsheehy@stjames.ie

**Keywords:** resource utilization, in-hospital mortality, emergency medical admissions

## Abstract

Demand for hospital resources may increase over time; we have examined all emergency admissions (51,136 episodes) from 2005 to 2013 for underlying trends and whether resource utilization and clinical risk are correlated. We used logistic regression of the resource indicator against 30-day in-hospital mortality and adjusted this risk estimate for other outcome predictors. Generally, resource indicators predicted an increased risk of a 30-day in-hospital death. For CT Brain the Odds Ratio (OR) was 1.37 (95% CI: 1.27, 1.50), CT Abdomen 3.48 (95% CI: 3.02, 4.02) and CT Chest, Thorax, Abdomen and Pelvis 2.50 (95% CI: 2.10, 2.97). Services allied to medicine including Physiotherapy 2.57 (95% CI: 2.35, 2.81), Dietetics 2.53 (95% CI: 2.27, 2.82), Speech and Language 5.29 (95% CI: 4.57, 6.05), Occupational Therapy 2.65 (95% CI: 2.38, 2.94) and Social Work 1.65 (95% CI: 1.48, 1.83) all predicted an increased risk. The in-hospital 30-day mortality increased with resource utilization, from 4.7% (none) to 27.0% (five resources). In acute medical illness, the use of radiological investigations and allied professionals increased over time. Resource utilization was calibrated from case complexity/30-day in-hospital mortality suggesting that complexity determined the need for and validated the use of these resources.

## 1. Introduction

The recent rising demand in healthcare services together with spiralling general healthcare costs has led to increased investigation into identifying service effectiveness [1,2]. Clinical services are under scrutiny in an attempt to achieve greater healthcare cost-efficiency and ultimately to try to reduce healthcare costs [3]. However, the past decade has also seen a significant increase in demand for investigations, particularly advanced radiology imaging (CT, MRI, PET and SPECT) [4]. MRI and CT examination numbers increased by 91% and 82% between 2000 and 2006 in England [5], while over the same period imaging services grew at more than twice the rate of physician services for certain sectors in the US [6]. The increased use of advanced radiology imaging for emergency department (ED) patients has not necessarily been reflected by a correlative increase in improved outcomes [7,8]. The question remains as to whether an increase in demand for resource may be associated with an increased benefit, or rather whether it leads to an increased length of stay, healthcare burden and exposure to ionising radiation [9]. Much of the cost of acute healthcare may be reflected in personnel costs, with resources such as physiotherapy, dietetics, occupational therapy and speech and language being required consequent on the demographics of the ageing population and rising demand for healthcare; the consumption pattern may change over time driven by societal or individual choices [10]. Furthermore, whether it is the change in the demographics combined with the complexity of medical needs of the ageing population that ultimately drives the increased demand for resource has been debated [11,12].

The aim of this study was first to examine the extent to which resource demand had increased over time and, second, to characterize the relationship between resource investigations (radiological or allied health professional demands) and clinical risk. It was hoped that utilization of services would associate with those lower acuity patients thus offering potential for resource savings. To this end we interrogated a database of all acute medical emergency admissions between 2005 and 2013 inclusive.

## 2. Methods

### 2.1. Background

St James’s Hospital (SJH) serves as a secondary care centre for emergency admissions for its local Dublin catchment area of 270,000 adults. Emergency medical patients are admitted from the Emergency Department to an Acute Medical Admissions Unit (AMAU), the operational details of which have been previously described [13,14,15]. Typically patients either arrive by ambulance (54%) or private transport with or without a referral letter from their General Practitioner. After initial assessment, patients deemed to possibly require admission to the hospital are referred to the team of the day (one of nine teams operating a 1:9 24 h on-call roster). The “on-call” system is covered by a “physician of the day” and the corresponding “team” (Senior Registrar/Registrar and Senior House Officer(s)); there was a post-call review round by the admitting Consultant on the following morning. Emergency medical patients were intended to be admitted from the Emergency Department to an Acute Medical Admissions Unit (AMAU) opened in 2003, under the care of a physician certified in General Internal Medicine and a subspecialty.

### 2.2. Data Collection

We employed an anonymous patient database assembling core information about each clinical episode from elements contained on the patient administration system, the national hospital in-patient enquiry (HIPE) scheme, the patient electronic record, the emergency room and laboratory systems. HIPE is a national database of coded discharge summaries from acute public hospitals in Ireland [16,17]. Ireland used the International Classification of Diseases, Ninth Revision, Clinical Modification (ICD-9-CM) for both diagnosis and procedure coding from 1990 to 2005 and ICD-10-CM since then.

Data held on the database includes the unique hospital number, admitting consultant, date of birth, gender, area of residence, principal and up to nine additional secondary diagnoses, principal and up to nine additional secondary procedures, and admission and discharge dates. Additional information cross-linked and automatically uploaded to the database includes physiological, haematological and biochemical parameters.

### 2.3. Study Inclusion Criteria

For this study, data was related to all emergency general medical patients admitted to SJH between 2005 and 2013. We considered each patient once only for the purposes of this assessment; if there was more than one admission, we used the last admission. Approximately 9.9% of our patients stay >30 days with a median LOS of 54.8 days (IQR 38.8, 97.2)—the majority of these are awaiting nursing home beds or other social interventions rather than ongoing admissions for medical reasons. We have therefore chosen a truncated end-point (death or episode completed by the 30-day endpoint) for analysis, to avoid the additional confounding of non-medical reasons for continuing as an inpatient.

### 2.4. Categorisation of Risk

We used known predictors of outcome to predict risk—these included Acute Illness Severity Score [18,19,20], Charlson Co-Morbidity Index [21], Chronic Disabling Disease score [22], Manchester Triage Category [23], and Sepsis Status. We previously described and validated an Acute Illness Severity Score based on serum sodium, potassium, urea, albumin, red cell distribution width, and white blood cell count. This has been used as a risk adjustor in our multiple variable model [18,19,20]. The underlying principle is that deviation beyond the bounderies of “normal homeostasis” is an estimate of risk, although the relationship is non-linear and differs for each variable. The frequency of illness severity groups 1 to 6 was 4.2%, 9.3%, 13.5%, 15.4%, 15.5 and 30.2% with a respective 30-day in-hospital mortality risk calculated by unique patient (last episode if >1) as 0.14%, 0.10%, 0.7%, 1.5%, 4.8% and 24.1% respectively.

The Charlson Co-Morbidity index provides a previously validated descriptor of the extent of Co-Morbidity [21]. Co-Morbidity is the presence of one or more additional disorders (or diseases) co-occurring with a primary disease or disorder. The Charlson Co-Morbidity index predicts the ten-year mortality outcomes for patients who may have a range of co-morbid conditions. Each condition is assigned a score of 1, 2, 3, or 6, depending on the mortality; scores are then summed into three classifying groups (Groups 0, 1 and 2). Charlson Co-Morbidity frequency of groups 0, 1 and 2 was 53.3%, 23.2% and 23.5% and 30-day in-hospital mortality rates of 3.0%, 8.7% and 21.8% respectively.

Disabling disease score has been demonstrated to be a predictor of mortality and therefore has been used as a risk adjustor in our multivariable model [22]. The frequency of patients with a chronic disabling score of 0, 1, 2, 3 or 4+ codes (1 point allocated for codes in one of 8 discrete disease categories) was 14.7%, 27.7%, 27.7%, 18.5% and 11.5% with respective 30-day in-hospital mortalities of 1.2%, 4.2%, 7.8%, 13.0% and 24.4%.

The urgency of each case presenting to the Emergency Department was assessed using the Manchester Triage Category and used as a risk adjustor [23]. Manchester Triage Categories of 1, 2 and 3 occurred with a frequency of 1.9%, 38.0% and 60.1% with respective 30-day in-hospital mortalities of 44.6%, 11.9% and 5.5%.

Sepsis (*i.e*., a septic screen), was categorized by no blood culture request (1), culture request but negative result (2) and culture request with positive result (3), and assessed for associated 30-day mortality risk. Sepsis was predictive of an in-hospital death by day 30 with categories 1, 2 and 3 present in 75.4%, 22.2% and 3.4% of patients, with respective in-hospital mortality rates of 2.9%, 8.9% and 18.2% by day 30. The Odds Ratios of a death for second and third categories (compared with no blood culture) were 3.2 (95% CI: 3.0, 3.5) and 7.3 (95% CI: 6.5, 8.3).

### 2.5. Statistical Methods

**Figure 1:**
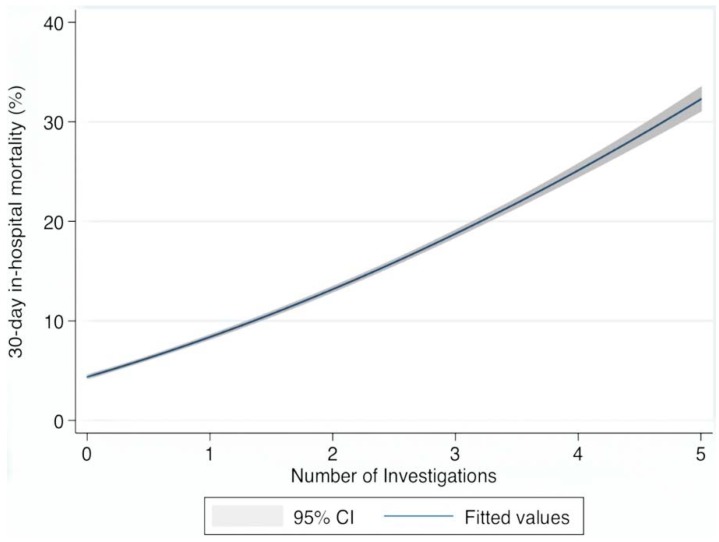
Utilizations of Services/Investigations and 30-day in-hospital mortality.

Descriptive statistics were calculated for background demographic data, including means/standard deviations (SD), medians/interquartile ranges (IQR), or percentages. Comparisons between categorical variables and mortality were made using chi-square tests. Candidate predictor variables (identified use of radiological or allied professional resource) were assessed as a univariable and subsequently adjusted by fitting a multi-variable logistic regression model for those parameters that were univariably predictive. The model parameters were stored; post-estimation intramodel and cross-model hypotheses could thereby be tested. The two way quadratic model fit (Figure 1) calculated the predicted probabilities from the logistic model and used the predicted values to derive the curve with the confidence intervals of the estimates.

Adjusted odds ratios (OR) and 95% confidence intervals (CI) or incidence rate ratios (IRR) were calculated for those predictors that significantly entered the model (*p* < 0.10). Statistical significance at *p* < 0.05 was assumed throughout. Stata v.13.1 (Stata Corporation, College Station, TX, USA) was used for analysis.

## 3. Results

### 3.1. Patient Demographics

A total of 51,136 episodes were recorded in 28,934 unique patients over the study period. These episodes represented all emergency medical admissions (including patients admitted directly into the Intensive Care Unit or High Dependency Unit) who had been discharged home or who had suffered an in-hospital death within 30 days of admission. The proportion of males and females were 48.2% and 51.8% respectively. The median (IQR) length of stay (LOS) was 4.4 (1.8, 8.9) days. The median (IQR) age was 58.0 (37.7, 75.9) years. The major disease categories (MDC) were respiratory (21.1%), cardiovascular (16.9%), neurological (19.5%), gastrointestinal (10.1%), hepatobilary (5.1%) and renal (4.9%). The characteristics of patients who survived or died in-hospital are shown in Table 1.

**Table 1:** Demographics of Emergency Medical Admissions (2005–2013).

Factor	Level	Survived	Died	*p*-Value
*N*		26646	2288	
Gender	Male	12873 (48.3%)	1068 (46.7%)	0.13
	Female	13773 (51.7%)	1220 (53.3%)	
Age (years), mean (SD)		55.2 (21.4)	75.5 (14.6)	<0.001
LOS (days), mean (SD)		6.4 (6.2)	9.3 (8.0)	<0.001
Illness Severity Grade	I	1309 (5.5%)	1 (<1%)	<0.001
	II	2801 (11.8%)	4 (0.2%)	
	III	3954 (16.7%)	23 (1.0%)	
	IV	4459 (18.8%)	53 (2.4%)	
	V	4305 (18.2%)	194 (8.8%)	
	VI	6866 (29.0%)	1930 (87.5%)	
Manchester Triage	3	14742 (55.3%)	658 (28.8%)	<0.001
	2	11551 (43.3%)	1368 (59.8%)	
	1	353 (1.3%)	262 (11.5%)	
Disabling Disease Score	0	3663 (18.1%)	1659 (10.3%)	<0.001
	1	7078 (35.0%)	3075 (19.2%)	
	2	5953 (29.4%)	4100 (25.6%)	
	3	2681 (13.3%)	3994 (24.9%)	
	4	858 (4.2%)	3210 (20.0%)	
Charlson Index	0	15392 (57.8%)	441 (19.3%)	<0.001
	1	6569 (24.7%)	644 (28.1%)	
	2	4685 (17.6%)	1203 (52.6%)	

### 3.2. Resource Utilization over Time (Table 2)

Whilst there were time related increases in resource utilization, these were not uniform. The demand for Dietetics (*p* = 0.6) and Occupational Therapy (*p* = 0.18) did not change over the 12 year under consideration; Social Services showed variable utilization without an overall time related trend. The increased use of Speech and Language (2.9% to 4.4%: *p* < 0.001) may be attributable to the introduction of a formal stroke service. There was an increased used of imaging survey in the event of clinical uncertainty (CT Thorax, Abdomen and Pelvis (2.3% to 5.0%: *p* < 0.001)) and increased MR Brain imaging (5.4% to 9.1%: *p* < 0.001).

**Table 2:** Resource Utilization with time.

Resource	2008	2011	2013	*p* <
CT Brain	28.3%	33.5%	30.2%	0.001
MR Brain	5.4%	6.8%	9.1%	0.001
CT Abdomen	2.5%	2.9%	3.0%	0.002
CT CTAP *	2.3%	4.9%	5.0%	0.001
Physiotherapy	19.4%	20.3%	24.9%	0.001
Dietetics	13.5%	13.3%	13.5%	0.6
Speech/Language	2.9%	4.8%	4.4%	0.001
Occupation Therapy	11.5%	11.2%	11.7%	0.18
Social Services	16.9%	15.6%	16.3%	0.004

* Chest, Thorax, Abdomen and Pelvis.

### 3.3. Resource Utilization as Outcome Predictors (Table 3/Table 4, Figure 1)

The overall mortality by episode between 2005 and 2013 was 5.4% (95% CI 5.2%–5.6%) and by unique patient 7.9% (95% CI 7.6%–8.2%). Calculated by patient, there was a 53% relative risk reduction (RRR) between 2005 and 2013, from 10.6% to 5.0%, yielding a number needed to treat (NNT) of 17.8.

In terms of prediction, most resource indicators predicted a univariate increased risk of an in-hospital death by day 30 (Table 3); however, MR Brain imaging predicted a lower risk of a 30-day in hospital death 0.54 (95% CI: 0.43, 0.68). Overall for these resource indicators, the in-hospital mortality risk increased proportionate to resource utilization in the univariate; allocation of 1 point to each of CT Brain and services including Physiotherapy, Dietetics, Occupational Therapy and Speech and Language (maximum possible score—5 points) resulted in the following resource /mortality relationship for 0 to 5 points of 4.7%, 7.2%, 15.7%, 19.5%, 23.7% and 27.0% respectively.

**Table 3:** Univariate logistic regression prediction of 30-day in-hospital mortality by procedure/service.

Investigation	OR	95% CI	*p* > |z|
CT Brain	1.37	1.27, 1.50	0.001
MR Brain	0.54	0.43, 0.68	0.001
CT Abdomen	3.48	3.02, 4.02	0.001
CT Chest/Abdo/Pelvis	2.50	2.10, 2.97	0.001
Physiotherapy	2.57	2.35, 2.81	0.001
Speech and Language	5.29	4.57, 6.05	0.001
Dietetics	2.53	2.27, 2.82	0.001
Occupational Therapy	2.65	2.38, 2.94	0.001
Social Services	1.65	1.48, 1.83	0.001

OR: odds ratio.

The risk estimates were then, in the multivariate model, adjusted for each resource indicatorusing Illness Severity Score [18,19,20], Charlson Co-Morbidity Index [21], Chronic Disabling Disease score [22], Manchester Triage Category [23] and Sepsis Status [24]. This resulted in major changes, as compared with the univariate risk estimates—Physiotherapy, Dietetics and Occupational therapy were no longer of prognostic significance in the adjusted model, as shown in Table 4. Social Services became a survival predictor OR 0.71 (95% CI: 0.62, 0.82) and Speech and Language had its OR risk estimate greatly reduced: OR 1.93 (95% CI: 1.62, 2.29). CT Brain was the only test that increased the risk estimate after adjustment: OR 1.82 (95% CI: 1.62, 2.04). MR Brain, after adjustment, showed an increase in the estimate of survival to hospital discharge: OR 0.63 (95% CI: 0.48, 0.83).

**Table 4:** Multiple variable logistic regression prediction of 30-day in-hospital mortality with procedure/service adjusted for other predictor.

Investigation	OR	95% CI	*p* > |z|
Acute Illness Severity	3.41	3.11, 3.74	0.001
Charlson Index	1.56	1.45, 1.67	0.001
Debilitating Disease	1.29	1.22, 1.37	0.001
Sepsis Status	2.06	1.91, 2.23	0.001
Manchester Triage Category	2.71	2.47, 2.98	0.001
CT Brain	1.82	1.63, 2.04	0.001
MR Brain	0.63	0.48, 0.83	0.001
CT Abdomen	2.80	2.35, 3.35	0.001
CT Chest/Abdo/Pelvis	1.53	1.25, 1.87	0.001
Physiotherapy	0.99	0.88, 1.13	0.98
Speech and Language	1.93	1.62, 2.29	0.001
Dietetics	1.03	0.09, 1.18	0.66
Occupational Therapy	1.12	0.96, 1.31	0.15
Social Services	0.71	0.62, 0.82	0.001

## 4. Discussion

This study demonstrated that, for acute medical illness, the use of radiological investigations and allied health services increased over time. Further, there was a strong correlation with clinical outcome and case complexity. The use of resources and services, were deemed proportionate to patient acuity, suggesting the scope for potential resource savings and efficiencies in an acute setting of the emergency medical admission may be limited. There has been considerable interest in health resource utilization (HRU) for improving patient outcome and efficiencies in the acute medical setting and in more general applications. For instance, Acute Care for Elders (ACE) units, which provide structured specialized care for older patients suffering acute illness, have demonstrated lower medical care costs, shorter LOS, and fewer readmissions [25]. In-hospital stroke units, conversely, have been reported to impose a high demand on resources, impacting considerably on health-care expenditure [26]. Alternative policies to such units, promoting early hospital discharge and home-based rehabilitation, have evidenced cost-savings without compromise of clinical outcome [27].

A number of utilization review (UR) tools have been proposed for identifying inefficiencies in acute hospital care; however, their assessment as compared with expert panel opinions have yielded a low level of validity [28]. An extensive overview of their role for acute medical admissions concluded that their validity could be criticized by virtue of their ignoring the heterogeneity of patients and the ability of a patient to actually benefit from hospital admission. Thus, the role of UR instruments as definite arbiters of individual hospital admissions has been deemed unlikely, thought their applicability as screening tools may be useful [29]. Clinical complexity has parameters that can be objectively defined such as the biochemical Acute Illness Severity Score [18,19,20], Charlson Co-Morbidity Index [21], Chronic Disabling Disease score [22], Manchester Triage Category [23] and Sepsis Status [24]. In this study we therefore attempted to calibrate the resource utilization against clinical complexity and consequent outcome, with the hypothesis that the aggregate demand for radiological procedures and allied health care professional input should correlate if the latter were appropriate and largely driven by clinical complexity. Put simply, clinical complexity should correlate with resource utilization. Were it not the case, the potential for resource savings may be indicated. This is the first study of its kind proposing to calibrate resource utilization against clinical risk.

It is expected that there will be a significant increased demand for healthcare in the Irish population over the coming years, stemming from a combination of demographic and epidemiological change [30], thus extolling the need for identifying service efficiencies. In order to meet this demand and, at a minimum, maintain the existing level of service it is expected that an increase of 8% per annum in service funding is necessary. The economic outlook in Ireland over the medium term is not conducive to substantial additional investment in healthcare and, thus, the same resources would require considerable strategic planning and substantial reorganization to provide a minimum service [30]. Indeed, the resource is not in actuality static but has declined to an extent that may not be appreciated. Between 2008 and 2014, the Irish Health Service budget was reduced by €4 billion, or in percentage terms by approximately 22% [31]. If the aforementioned 8% annual increment to the 2008 budget of €14.7 billion is applied, with a 2%–3% inflation adjustment, an exchequer funding requirement of €27.6 billion in 2014 compared with the actual figure of €13.4 billion would be indicated.

It is against this backdrop of severe resource constraint, and having established increased resource utilization over the timeframe of our study, that we have sought to devise a model correlating clinical risk with service utilization. Any potential to reduce resource utilization might require that the latter were not driven by factors such as demography, illness severity, chronic disabling disease and co-morbidities, but perhaps by patient expectations or variations in clinical practice unrelated to the underlying disease. The majority of the services investigated, when analysed as a univariate predictor, were shown to correlate and predict the risk by day 30 of an in-hospital death. The implication is that the resource utilization was proportionate to and driven by the clinical risk—devising a summary metric, (the additive relationship of resources utilized per episode to 30-day mortality rates) showed excellent correlation. Hence, as this summary metric correlated with clinical risk, there did not appear to be any obvious capacity to limit the extent of in-hospital investigations.

When assessed using multivariate logistic analysis, however, the predictive weightings of services were demonstrated to differ considerably from their respective univariate analyses. The use of physiotherapy, dietetics and occupational therapy, after the multi-variate adjustment, were not predictive of clinical outcome. This is what would be anticipated, as per the univariate model, when one predictor parameter is a proxy for others in the model, such as illness severity, chronic debilitating disease or co-morbidities. Conversely, radiology examinations and speech and language therapy remained as significant outcome predictors even after adjustment using the multivariate model, showing strong association with those more complicated episodes. In particular, CT brain, the most utilized of the services assessed, was the only procedure to demonstrate an increased significance as a mortality predictor. The question has previously been raised in the literature as to whether the growing demand in advanced radiology imaging for emergency patients has correlated with improved outcomes [7,8] or rather led to an increased service burden [9]. In this context, our results suggest CT examinations, even when adjusted for illness severity, proved to be a strong predictor of mortality. Thus, CT brain is an intrinsically important investigation, as would be expected, that designates patients as likely to have a worse outcome when adjusted for other predictor variables.

Contrary to CT brain, MR brain was identified as predictive of survival in the multivariate model, an unexpected result given previous analysis at this centre identified an MR investigation as a mortality predictor [32]. The difference may highlight a limitation of national truncated datasets, such as HIPE. All hospital procedures per episode are not necessarily captured, with particular emphasis being placed on higher cost procedures—as the HIPE codes may have a secondary role as a hospital resource allocation model. Indeed, our previous MR study [32], which analyzed data from the Radiology Information System (RIS) and EPR, proved MRI to associate with higher mortality especially in patients with elevated illness severity (in the 4th and 5th quintiles). For these complex cases, which would expectedly incur higher costs, it is not unreasonable to suggest that MR exams may not have been captured correctly. This work focused on MR brain exams in particular while the previous MR study did not discriminate between exam type [32]. It is also noted at SJH that CT examinations are available on a 24 h basis while the MRI service is limited to weekdays between 9 am–5 pm. It is, thus, more likely that an emergency patient will undergo the former imaging investigation. Further, MRI scans require more complicated preparation and, due to their scan time length, may be deemed unsuitable for acutely ill patients. CT examinations, conversely, offer as a safer option. This is reflected in the MR patient cohort who present as younger and predominate in the lower acute illness severity categories, suggesting a less critical cohort than those associated with CT.

In recent years economic constraints have prompted a reduction in resources across healthcare providers; nevertheless, this study reports an increasing demand on radiology and allied services and, further, a significant reduction in mortality. This improved outcome can be attributed to the establishment of SJH’s AMAU which has improved healthcare delivery to emergency medical admissions [13,14,15]. The models have established those most acute patients to be in most need of services/resources leaving little option for savings. The identified predictors of poor outcome have been affirmed as necessary resources provided to the most unwell. In order to continue with such increased effectiveness, with a service coming under increasing pressure, additional capacity is required. While the hospital service is currently doing more with less, should further resources remain unprovided there may come a critical point after which improved outcomes will not be attainable.

As with any study, ours posseesses both strengths and limitations. One such strength lies in the large number of patients and the extended duration of the observations. In addition, all patients admitted as general medical emergencies were included, increasing the generalisability of our results, capturing those most acutely ill and stable patients and, thus, reflecting real world clinical practice. One limitation to be considered lies in the amount of detail provided by the HIPE datasets. For those more complex episodes, the level of detail may not be sufficiently granular. In addition, the hospital has a number of other admitting services, most notably cardiology under which acute coronary syndromes are admitted, which may limit the generalizability of the results.

## 5. Conclusions

This study has evaluated the demand for hospital resources in all emergency medical admissions over an 8-year period, showing there to be an increased use of radiological investigations and allied professional services. The correlation between resource indicators, clinical risk and 30-day in hospital death was examined with utilization of services shown to correlate with clinical complexity. Furthermore, the need for an increased number of resources associated with those more complex cases, as should be the case if complexity is reflected by utilisation. Our data suggests that the use of services is driven by clinical complexity, thus validating the need for such services at this centre. Those more acute patients use a proportionate amount of resources as justified by their acuity. With healthcare facilities under increased pressure as a result of funding cuts and increasing demand, the level of service provided will not suffice should resources remain at the current level. The devised generalizable models, applicable to sites with differing demographics, would present as useful identifiers of resource savings.
